# Low TAS1R2 Sweet Taste Receptor Expression in Skeletal Muscle of Genetically Diverse BXD Mice Mirrors Transcriptomic Signatures of Loss-of-Function Mice

**DOI:** 10.3390/nu17111918

**Published:** 2025-06-03

**Authors:** Kendall King, Joan Serrano, Nishita N. Meshram, Mahdiye Saadi, Lynn Moreira, Evaggelia G. Papachristou, George A. Kyriazis

**Affiliations:** Department of Biological Chemistry & Pharmacology, College of Medicine, The Ohio State University, Columbus, OH 43210, USA

**Keywords:** Tas1r2, T1r2, transcriptomics, skeletal muscle, BXD panel mice, microarray, metabolism, mitochondria, ribosomes, signaling, high fat diet, sweet taste receptors

## Abstract

**Background/Objectives:** Sweet taste receptor TAS1R2 is expressed in skeletal muscle, yet its role in muscle metabolism remains poorly understood. **Methods:** Here, we leverage the BXD recombinant inbred mouse panel and Tas1r2 whole-body knockout (bKO) models to investigate the transcriptional impact of Tas1r2 deficiency on skeletal muscle function. **Results:** A gene network analysis revealed significant overlap in transcriptomic signatures between BXD strains with low Tas1r2 expression (BXD L_Tas1r2_) and bKO muscle, particularly in pathways regulating oxidative phosphorylation, cytoplasmic ribosome function, and proteostasis. Notably, Tas1r2 expression negatively correlated with genes involved in fatty acid metabolism, suggesting its role in lipid utilization. Under high-fat diet (HFD) conditions, BXD_HFD_ L_Tas1r2_ mice exhibited further enrichment in pathways linked to proteasome degradation, oxidative stress, and interleukin signaling, amplifying the transcriptomic convergence with bKO models. Key transcription factors (Mlxipl, Nfic, Rxrb) exhibited altered regulatory patterns under dietary stress, indicating that TAS1R2 influences metabolic adaptability through transcriptional reprogramming. **Conclusions:** Given that human TAS1R2 variants rarely result in complete loss of function (LOF), the BXD panel provides an effective dose-dependent model to bridge the gap between knockout phenotypes and human SNP carriers. Our findings establish TAS1R2 as a metabolic regulator in skeletal muscle and highlight the utility of genetically diverse mouse populations in dissecting gene-diet interactions relevant to human metabolic diseases.

## 1. Introduction

Gene loss-of-function (LOF) is commonly studied through two primary approaches: naturally occurring human single nucleotide polymorphisms (SNPs) and laboratory-generated mouse knockout models. Human LOF SNPs often lead to partial functional impairment, providing insights into gene redundancy, evolutionary adaptation, and population-level variability [1,2]. In contrast, mouse knockout models typically induce complete gene inactivation in controlled genetic backgrounds, allowing researchers to dissect gene function and model human disease [3,4].

A key challenge arises when knockout phenotypes in mice do not fully recapitulate those observed in human LOF SNP carriers. Partial and complete LOF variants can lead to distinct phenotypic outcomes, and species-specific differences in gene regulation, genomic context, and compensatory mechanisms further complicate comparisons [4,5,6]. Additionally, human SNPs occur within outbred populations, whereas knockout models are often studied in inbred strains, highlighting the need for models that integrate both genetic diversity and graded functional loss.

The BXD recombinant inbred (RI) panel, derived from C57BL/6J and DBA/2J mouse strains, provides such a model. As the largest and most extensively characterized genetic reference population (GRP), consisting of ~160 isogenic strains, the BXD panel captures a broad range of natural genetic variation while preserving experimental reproducibility [7]. Unlike traditional RI panels, BXD panel strains exhibit increased recombination events, making them more genetically diverse and more representative of human populations [7,8].

BXD panel mice have been widely used in systems genetics to study complex traits, including metabolism, behavior, and molecular regulation [9]. Public databases such as GeneNetwork.org facilitate quantitative trait locus (QTL) mapping and genome-wide association studies (GWAS) within this panel, enabling researchers to identify genetic determinants of metabolic and other complex disorders [8,10]. By mimicking human genetic diversity while retaining the experimental control of traditional mouse models, the BXD panel provides a powerful platform to investigate gene function across a continuum of expression levels, paralleling the graded LOF effects often seen in human SNP carriers [8,11,12].

Here, we use the TAS1R2 gene to demonstrate the utility of the BXD panel in bridging the gap between human LOF SNPs and clonal knockout models. The TAS1R2 gene encodes the sweet taste receptor, a G-protein-coupled receptor (GPCR) implicated in taste sensing on the tongue [13,14] but also in peripheral nutrient metabolism [15]. For instance, studies using whole-body Tas1r2 knockout (bKO) C57BL/6J mice have revealed significant roles in insulin secretion [16,17] and GLP-2 induced intestinal glucose absorption [18]. Additionally, when fed a high-fat diet (HFD), bKO mice displayed improved body composition, including reduced fat mass, increased lean mass, lower fasting glucose levels, and reduced hepatic triglyceride accumulation [19], suggesting a broader role for TAS1R2 in peripheral nutrient sensing under metabolic stress.

Notably, several metabolic associations have also been tested and observed in humans with the TAS1R2 Ile191Val (rs35874116) polymorphism (TAS1R2^Ile191Val^) [20,21,22,23,24,25,26,27] which results in partial LOF due to reduced receptor complex membrane stability [28,29,30]. Carriers of TAS1R2^Ile191Val^ exhibited reduced glucose excursions during an oral glucose challenge [28], similar to bKO mice [18]. The impact of TAS1R2^Ile191Val^ on glucose metabolism was further confirmed in two independent studies, showing that individuals with this partial LOF variant had lower glycosylated hemoglobin (HbA1c) [31,32], a key measure of glucose homeostasis. Moreover, carriers of TAS1R2^Ile191Val^ displayed improved muscle mass, mitochondrial function, and endurance following an exercise training intervention [31], suggesting a potential role of TAS1R2 in skeletal muscle metabolism. Indeed, muscle-specific Tas1r2 deletion (mKO) in mice recapitulated these human phenotypes, demonstrating enhanced mitochondrial function and endurance [33].

Although both mKO and bKO models exhibit overlapping muscle and systemic phenotypes, they represent complete TAS1R2 LOF, which may not fully align with the partial LOF seen in human SNP models. The BXD mouse panel addresses this limitation by offering a spectrum of Tas1r2 expression levels across strains. By leveraging public transcriptomic datasets from GeneNetwork.org and comparing them to in-house global knockout transcriptomic data, we aim to identify overlapping molecular signatures. This approach enables the validation of key pathways associated with Tas1r2 LOF in a genetically diverse context, where the BXD panel more accurately reflects human population variability. Ultimately, this strategy enhances the translational relevance of TAS1R2 research and provides a scalable framework to study LOF effects.

## 2. Materials and Methods

### 2.1. Animal Model and Tissue Collection

Whole-body TAS1R2 knockout (BL6 KO_Tas1r2_) mice, generously provided by Dr. Zuker [34], were maintained in accordance with protocols approved by The Ohio State University Institutional Animal Care and Use Committee (IACUC), Ethic approval code: 2018A00000039-R1, Ethic approval date: 21 April 2021. BL6 KO_Tas1r2_ mice were housed under a 12-h light/dark cycle with unrestricted access to water and a standard diet. Males were used for experiments at 12–13 weeks of age after sexual maturity. Muscles were harvested from 5 h fasted male BL6 wildtype (WT_Tas1r2_, n = 3) and BL6 KO_Tas1r2_ mice (n = 3), washed in ice-cold phosphate-buffered saline (PBS), and immediately flash-frozen in liquid nitrogen to preserve RNA integrity. Frozen samples were pulverized and homogenized in a cold lysis buffer (20 mM Tris-HCl pH 7.4, 10 mM MgCl, 200 mM KCl, 2 mM DTT, 1% Triton X-100; all reagents were from Invitrogen-Thermo Fisher Scientific, Waltham, MA, or Millipore-Sigma, Burlington, MA, USA) using a Dounce homogenizer (Wilmad, Vineland, NJ, USA). The homogenates were transferred to RNase-free tubes, and centrifuged at 17,000× *g* at 4 °C for 10 min, and the resulting supernatant was used for RNA preparation.

### 2.2. RNA Isolation and Analysis

Total RNA was extracted from the homogenized BL6 muscle samples using TRIzol reagent (Invitrogen-Thermo Fisher Scientific, Waltham, MA, USA) according to the manufacturer’s protocol. Following TRIzol extraction, RNA was purified using a commercial RNA purification kit (Zymo Research, Irvine, CA, USA) to ensure high purity and yield. The quality and quantity of RNA were assessed using an Agilent TapeStation 4200 (Agilent, Santa Clara, CA, USA) and a NanoDrop spectrophotometer (Thermo Fisher Scientific, Waltham, MA, USA). The RNA samples meeting our quality control criteria were used for transcriptomics.

### 2.3. Transcriptomics

Skeletal muscle transcriptomics data from 29-week-old male BXD panel mice were sourced from GeneNetwork.org (EPFL/LISP BXD CD+HFD Muscle Affy Mouse Gene 1.0 ST (Nov12) RMA Exon Level). Detailed methods regarding the phenotyping and tissue collection for these datasets were previously described [35] and additional information can be found in the metadata summary page provided by GeneNetwork.org (https://info.genenetwork.org/infofile/source.php?GN_AccesionId=395, accessed 28 May 2025). The RNA samples derived from BL6 WT_Tas1r2_ and BL6 KO_Tas1r2_ muscles passedthe quality control (RNA Integrity Numbers (RIN) exceeded 8.0) and were submitted to the Ohio State University Genomics Core for transcriptomic profiling using the Affymetrix Mouse Clariom S hybridization array (Thermo Fisher Scientific, Waltham, MA). The samples were processed according to the manufacturer’s guidelines for the Clariom S Pico assay, which included reverse transcription, amplification, and labeling. The hybridized arrays were scanned to produce expression data, and raw intensities were analyzed using Affymetrix Transcriptome Analysis Console (TAC) software with default settings for the Clariom S Mouse transcriptome version 2.

### 2.4. Comparative Transcriptomics in BL6 and BXD Models

BL6 KO_Tas1r2_ microarray data, previously log_2_ transformed, was further normalized to match the processing applied to the BXD panel datasets from GeneNetwork.org (https://genenetwork.org/, accessed 28 May 2025). This included global z-score normalization, followed by scaling to a mean of 8 with a standard deviation of 2. Re-normalizing the BL6 WT_Tas1r2_ and BL6 KO_Tas1r2_ data ensured a more robust and statistically sound comparison with the BXD panel dataset [36,37].

### 2.5. Gene Set Enrichment and Overrepresentation Analysis

A Gene Set Enrichment Analysis (GSEA) was performed with the fgsea package [38] in R (R version 4.3.3) and a reference-level comparison matrix to compare transcriptomic signatures between BL6 and BXD panel models. Enrichment scores and *p*-values were calculated using Kolmogorov-Smirnov tests [39], and significance was adjusted with Benjamini-Yekutieli (BY) corrections [40]. Additionally, an over-representation analysis (ORA) was conducted through the WEB-based Gene SeT AnaLysis Toolkit (WebGestalt 2024) [41]. Both GSEA and ORA were applied to WikiPathways to identify functionally enriched gene sets and provide insights into shared transcriptomic signatures across models [42].

### 2.6. Statistics

Statistical analyses were performed using R (version 4.3.3) and Prism GraphPad (version 9.0) [43]. T-tests were conducted to compare gene expression levels between the BXD H_Tas1r2_ and BXD L_Tas1r2_ groups, with the *p*-values adjusted for multiple comparisons using the Benjamini–Yekutieli (BY) method to control the false discovery rate (FDR; adjusted *p* < 0.05). A Pearson’s correlation analysis identified genes significantly associated with Tas1r2 expression in the BXD panel dataset (n = 42), while a correlation analysis was not performed for the BL6 groups (n = 3) due to insufficient sample size. Figures were generated in R and Prism GraphPad.

## 3. Results

### 3.1. Tas1r2 Expression Ranking and Correlation Analysis in Skeletal Muscle of Genetically Diverse BXD Mice

**Figure 1:**
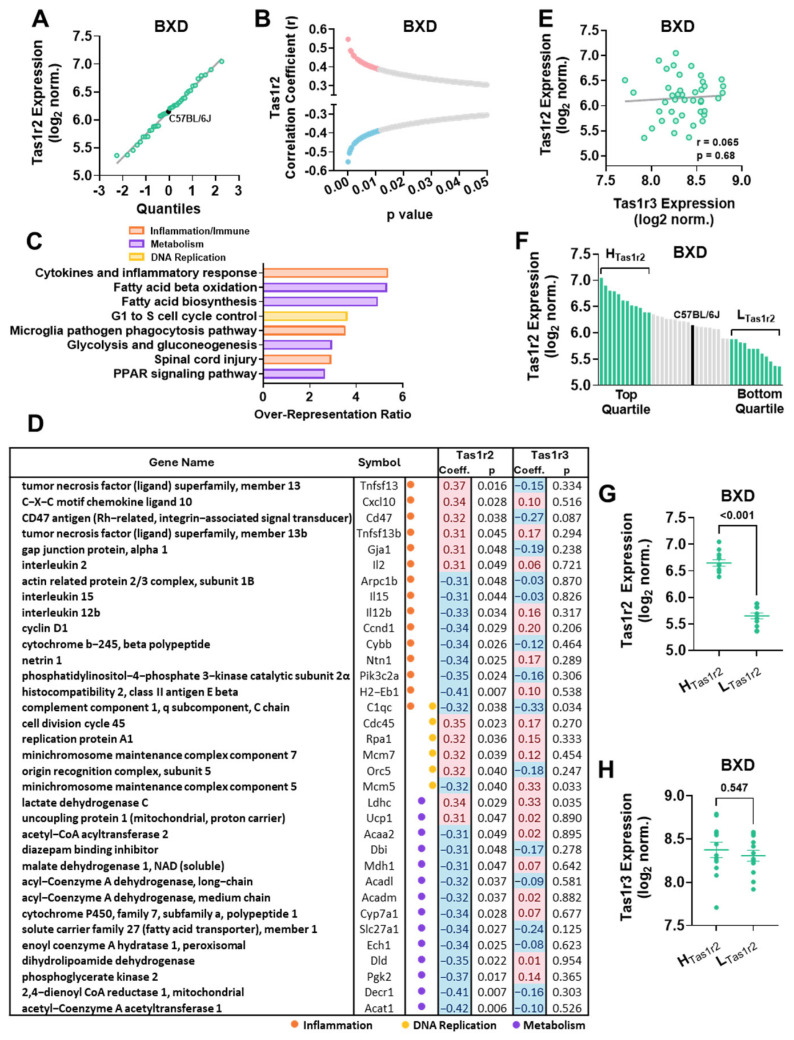
Tas1r2 expression ranking and correlation analysis in skeletal muscle of BXD mice. (**A**) Normal probability plot of Tas1r2 expression across skeletal muscles of BXD mice (n = 42). C57BL/6J mouse strain is highlighted (black dot) as reference. (**B**) Statistically significant (*p* < 0.05) Pearson’s correlation coefficients (r) between gene expression in skeletal muscle and Tas1r2 (n = 613). Most significant (*p* < 0.01) positive (red) and negative (blue) correlations are highlighted. (**C**) Over-Representation Analysis (ORA) of highly correlated genes to pathways (Wikipathways) grouped by functional categories. (**D**) Non-redundant list of genes contributing to the ORA in (**C**), showing r (positive in red and negative in blue) and *p*-values for Tas1r2 and Tas1r3 gene associations. Color dots show gene grouping according to ORA pathway categories shown in (**C**). (**E**) Correlation between Tas1r2 and Tas1r3 expression in skeletal muscles of BXD mice (n = 42). (**F**) Tas1r2 expression rank in skeletal muscles of BXD panel mice. Mice (green) stratified into high Tas1r2 expression (H_Tas1r2_, n = 12, top quartile) and low Tas1r2 expression (L_Tas1r2_, n = 12, bottom quartile) groups. C57BL/6J mouse strain is shown in black. (**G,H**) Tas1r2 and Tas1r3 expression levels between H_Tas1r2_ and L_Tas1r2_ BXD panel groups. T-test *p*-value.

We accessed skeletal muscle mRNA expression data from the GeneNetwork.org dataset, ‘EPFL/LISP BXD CD Muscle Affy Mouse Gene 1.0 ST (Nov12) RMA Exon Level’, which includes eight probes for Tas1r2. We selected Probe ID: 10509812 for analysis due to its lack of cross-hybridization, normal expression distribution, and broad dynamic range across mouse strains (Figure 1A, Supplemental File S1A). To explore potential regulatory networks, we performed an unbiased correlation analysis between Tas1r2 expression and 38,494 gene targets. This revealed 613 genes significantly correlated with Tas1r2 expression (*p* < 0.05) in chow-fed BXD panel mice (Figure 1B, Supplemental File S1B). The over-representation analysis (ORA; WebGestalt 2024 [41]) identified enrichment in pathways related to the inflammation/immune response, nutrient metabolism, and DNA replication (Figure 1C, Supplemental File S1C). Notably, 12 genes involved in fatty acid metabolism were all negatively correlated with Tas1r2 expression, suggesting a link between Tas1r2 signaling and lipid metabolism in muscle (Figure 1D).

Since Tas1r2 forms an obligate heterodimer with Tas1r3 [13], we assessed the correlation between Tas1r2 and Tas1r3 expression. We found no significant correlation between the two genes (Figure 1E), despite higher overall Tas1r3 expression, as previously reported [33]. Additionally, Tas1r3 expression did not correlate with the gene set identified in the Tas1r2 ORA analysis (Figure 1D, right), confirming that these transcriptomic associations are specific to Tas1r2 expression rather than general sweet taste receptor function.

### 3.2. Gene Set Enrichment Analysis (GSEA) in BXD Mice Stratified by Tas1r2 Expression

**Figure 2:**
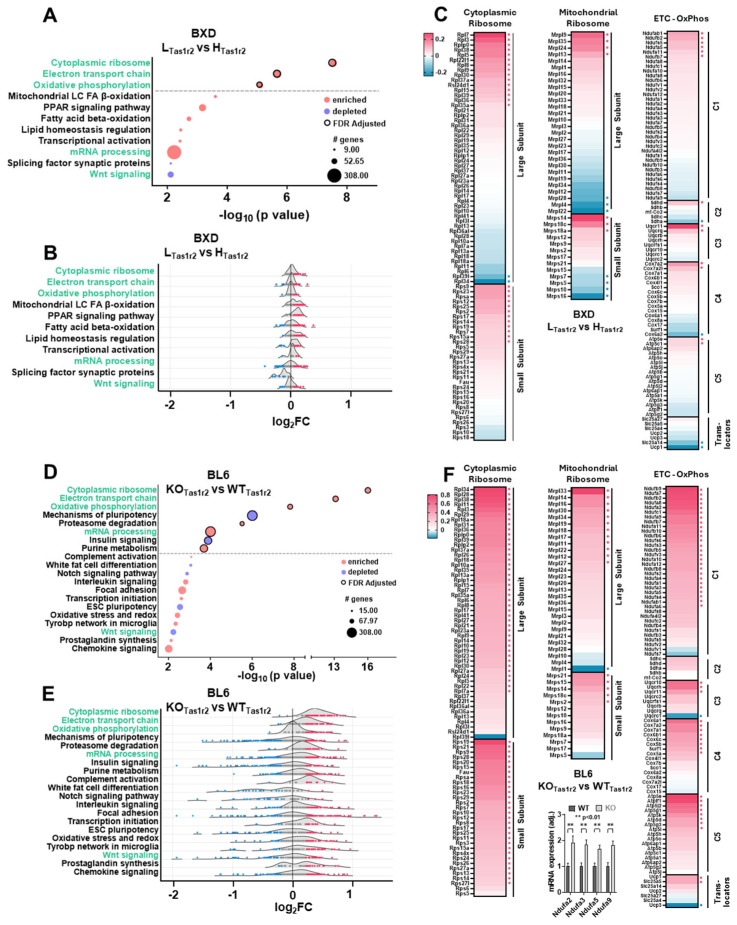
Gene Set Enrichment Analysis (GSEA) in BXD mice stratified by Tas1r2 expression. (**A**,**D**) GSEA (Wikipathways) in BXD L_Tas1r2_ ((**A**), n = 12) and BL6 KO_Tas1r2_ ((**D**), n = 3) muscles. Common pathways between BXD panel and BL6 analyses are highlighted in green. (**B**,**E**) Individual genes ranked by log_2_ fold change (log_2_FC) (average normalized L_Tas1r2_—average normalized H_Tas1r2_; average normalized KO_Tas1r2_—average normalized WT_Tas1r2_) are represented as dots for each pathway ridgeline curve, with highly-ranking genes highlighted as either enriched (red) or depleted (blue). (**C**,**F**) Heatmaps of gene log_2_FC values for top 3 FDR-corrected common pathways showing distribution of highly-ranked genes (red or blue dots) in BXD L_Tas1r2_ (**C**) or BL6 KO_Tas1r2_ (**F**) muscles. (**F** inset) qPCR of mitochondiral gene markers in BL6 WT_Tas1r2_ (n = 7) and KO_Tas1r2_ (n = 7) muscles.

To investigate whether variability in Tas1r2 expression leads to distinct transcriptomic signatures in muscle, we ranked Tas1r2 expression across 42 BXD panel strains and observed a 3.22-fold difference between the highest- and lowest-expressing strains (Figure 1F, Supplemental File S1D). C57BL/6J (BL6) mice ranked at the 49th percentile, providing a reference point for common laboratory strains. We stratified BXD panel strains into high Tas1r2 (H_Tas1r2_, top quartile, n = 12) and low Tas1r2 (L_Tas1r2_, bottom quartile, n = 12) groups (Figure 1F), confirming significant differences in Tas1r2 expression between the two groups (Figure 1G). Tas1r3 expression remained unchanged between the groups (Figure 1H), reinforcing that observed transcriptomic differences reflect Tas1r2 expression variability rather than Tas1r3-mediated effects. The GSEA analysis between the BXD L_Tas1r2_ and BXD H_Tas1r2_ groups identified the significant enrichment of pathways related to ribosomal proteins (protein synthesis), mitochondrial function, and lipid metabolism in L_Tas1r2_ muscles (Figure 2A,B, Supplemental File S1E). To further dissect the data, we generated heatmaps of key enriched pathways, distinguishing between cytoplasmic and mitochondrial ribosomal proteins and consolidating electron transport chain (ETC) and oxidative phosphorylation (OxPhos) pathways (Figure 2C, Supplemental File S1F). A gene-level analysis revealed key drivers of these pathways, including Rpl7, Rpl3, Rps23 (cytoplasmic ribosome), Mrpl9, Mrps14, Mrps16 (mitochondrial ribosome), Uqcr11, Cox7a2, Ndufab1, and Ucp1 (ETC-OxPhos).

### 3.3. Comparison of Muscle Transcriptomics Between BXD L_Tas1r2_ and BL6 KO_Tas1r2_ Mice

The transcriptomic signatures observed in BXD L_Tas1r2_ muscle closely align with phenotypes of Tas1r2-deficient mice, which exhibit increased mitochondrial density, enhanced endurance, and higher lean mass [19,31,33]. To determine whether low Tas1r2 expression in BXD panel mice mirrors the complete genetic deletion of Tas1r2, we compared muscle transcriptomes between BL6 WT_Tas1r2_ (wildtype) and BL6 KO_Tas1r2_ (knockout) mice. GSEA analysis confirmed significant enrichment in cytoplasmic ribosomal proteins, ETC, and OxPhos pathways in BL6 KO_Tas1r2_ compared to BL6 WT_Tas1r2_ muscle (Figure 2D, Supplemental File S1G). Notably, these enriched pathways are identical to those observed in BXD L_Tas1r2_ muscle, strengthening the functional link between Tas1r2 loss and mitochondrial protein synthesis enhancement. However, the BL6 KO_Tas1r2_ muscles exhibited additional transcriptional alterations, including pluripotency pathways, proteasome degradation, insulin signaling, and purine metabolism, suggesting that complete Tas1r2 loss leads to broader regulatory effects beyond those seen in naturally low-expressing strains. The gene-level analysis showed a comparable but broader distribution of highly ranked genes in BL6 KO_Tas1r2_ and BXD L_Tas1r2_ muscle (Figure 2E,F, Supplemental File S1H), reinforcing mitochondrial function and protein synthesis as core transcriptomic shifts associated with Tas1r2 deficiency. The up-regulation of the mitochondrial genes of the ubiquinone oxidoreductase subunit Ndufa2, Ndufa3, Ndufa5, and Ndufa9 was confirmed in BL6 KO_Tas1r2_ muscle (Figure 2F inset).

### 3.4. Transcriptional Regulation of Shared Molecular Signatures in BL6 KO_Tas1r2_ and BXD L_Tas1r2_ Mice

**Figure 3:**
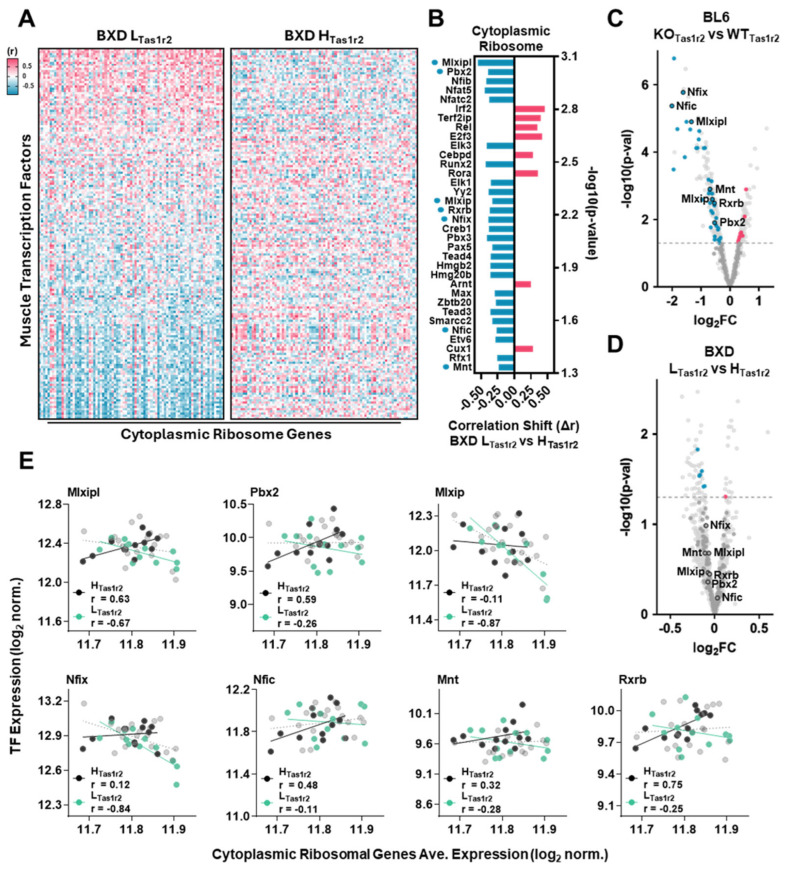
The transcriptional regulation of cytoplasmic ribosome in BL6 KOTas1r2 and BXD L_Tas1r2_ muscles. (**A**) Heatmaps of the Pearson’s correlation coefficient (r) values between muscle-specific transcription factors (TFs) (n = 168) and cytoplasmic ribosomal protein genes (average of n = 81), sorted by the average r for each TF in the BXD L_Tas1r2_ group. (**B**) The differences in the average r between muscle-specific TFs and cytoplasmic ribosomal proteins genes (average of n = 81) in the BXD L_Tas1r2_ and BXD H_Tas1r_2 mice. Correlation shifts (Δr) are represented as negative shifts (blue) or positive shifts (red). The blue dot next to the TF name indicates common genes between BXD and BL6 group comparisons (shown in panel (**C**,**D**). (**C**,**D**) A volcano plot of the relative expression of TF (n = 1094) in the BL6 (n = 3) or BXD (n = 12) groups. The horizontal dotted line shows the threshold of statistical significance (*p* < 0.05). Statistically significant muscle-specific TF expression is shown in blue (depleted) or red (enriched). Non-significant muscle-specific TF expression is shown in dark gray. Overlapping genes between BL6 and BXD panel groups are circled and labeled. (**E**) Correlations between muscle-specific TF expression (from panels (**C**,**D**)) and the average cytoplasmic ribosomal gene (n = 81) expression in BXD L_Tas1r2_ BXD H_Tas1r2_ (black) and BXD L_Tas1r2_ (green) muscles. All the remaining BXD panel strains (e.g., not included in H_Tas1r2_ or L_Tas1r2_ groups) are shown in gray.

To identify transcription factors (TFs) driving these overlapping transcriptomic signatures, we performed correlation analysis of TFs in BXD L_Tas1r2_ vs. H_Tas1r2_ muscle (Figure 3A). Pearson’s correlation analysis of muscle-specific TFs [44] and key GSEA pathways revealed significant regulatory shifts (Supplemental File S1I). Among 34 TFs exhibiting significant shifts in BXD L_Tas1r2_ muscle, 26 were negatively correlated with cytoplasmic ribosomal genes (Figure 3B). Comparative analysis in BL6 KO_Tas1r2_ vs. WT_Tas1r2_ muscles confirmed a greater number of differentially expressed TFs (Figure 3C, Supplemental File S1J), indicating exacerbated transcriptional differences with complete Tas1r2 loss. Notably, seven TFs (Mlxipl, Pbx2, Mxlip, Rxrb, Nfix, Nfic, and Mnt) were negatively correlated with cytoplasmic ribosomal genes in BXD L_Tas1r2_ muscle and significantly downregulated in BL6 KO_Tas1r2_ muscle, reinforcing their functional role in Tas1r2-mediated transcriptional regulation (Figure 3D,E, Supplemental File S1J).

No significant shifts in TF correlations or overlaps in expression patterns were observed for the mitochondrial ribosome (Supplemental File S1I). We extended our analysis to the ETC–OxPhos pathway (Figure S1A) and identified 41 TFs with significant correlation shifts between the BXD L_Tas1r2_ and BXD H_Tas1r2_ groups, with 27 exhibiting negative shifts (Figure S1B). Among these, seven TFs (Nfic, Mlxipl, Foxk2, Sim2, Rxrb, Pbx2, and Rreb1) displayed overlapping differential expression patterns in both the BL6 KO_Tas1r2_ (Figure S1C) and BXD L_Tas1r2_ (Figure S1D) datasets, along with pronounced correlation shifts between BXD L_Tas1r2_ and BXD H_Tas1r_2 muscles (Figure S1E). Notably, Mlxipl, Nfic, Rxrb, and Pbx2 were commonly associated with both the cytoplasmic ribosome and ETC–OxPhos pathways, suggesting their involvement in regulating these interconnected processes.

### 3.5. The Effects of a High-Fat Diet (HFD) on the Transcriptional Signatures in BXD Muscles

**Figure 4:**
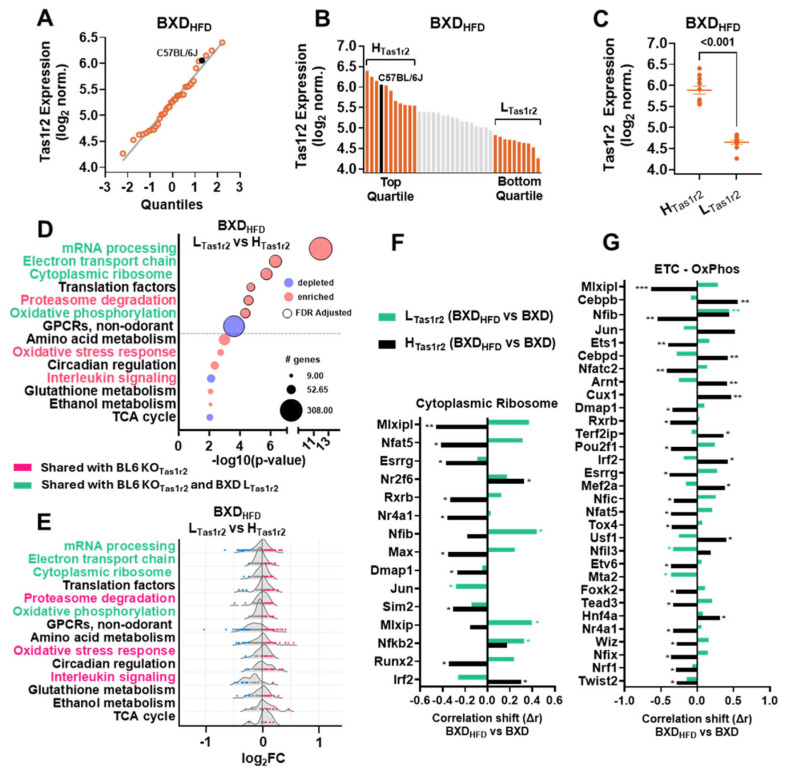
Effects of high-fat diet (HFD) on the transcriptional signatures in BXD panel muscles. (**A**) Normal probability plot of Tas1r2 expression across skeletal muscles of BXD_HFD_ mice (n = 37). C57BL/6J mouse strain is highlighted (black dot) as reference. (**B**) Tas1r2 expression rank in skeletal muscles of BXD_HFD_ mice. Mice (green) stratified into high Tas1r2 expression (H_Tas1r2_, n = 11, top quartile) and low Tas1r2 expression (L_Tas1r2_, n = 10, bottom quartile) groups. C57BL/6J mouse strain is shown in black. (**C**) Tas1r2 levels between H_Tas1r2_ and L_Tas1r2_ BXD_HFD_ groups. T-test *p*-value. (**D**) GSEA (Wikipathways) in BXD_HFD_ L_Tas1r2_. Common pathways between BXD_HFD_ and both BXD and BL6 analyses are highlighted in green, and pathways common only between BXD_HFD_ and BL6 analyses are highlighted in red. (**E**) Genes are ranked by log_2_FC (average normalized L_Tas1r2_—average normalized H_Tas1r2_) and represented as dots for each Wikipathway’s ridgeline curve, with highly-ranking genes highlighted as either enriched (red) or depleted (blue) for BXD_HFD_ L_Tas1r2_. (**F**,**G**) Effects of high-fat diet (HFD) on the differences in average r between muscle-specific TFs and cytoplasmic ribosomal protein genes (average of n = 81) or ETC-OxPhos genes (average of n = 89) in BXD L_Tas1r2_ and BXD H_Tas1r2_ mice. Correlation shifts (Δr) are represented as negative shifts (black) or positive shifts (green). ETC-OxPhos, electron transport chain-oxidative phosphorylation. * *p* <0.05, ** *p* < 0.01, *** *p* < 0.001.

Since Tas1r2 deletion in BL6 mice protects against HFD-induced metabolic dysfunction [19], we assessed how HFD alters muscle transcriptomic signatures in BXD panel mice. We analyzed the GeneNetwork.org dataset ‘EPFL/LISP BXD HFD Muscle Affy Mouse Gene 1.0 ST (Nov12) RMA Exon Level.’ using the TAS1R2 probe 10509812 (Figure 4A). We stratified the BXD_HFD_ mice (n = 37) into H_Tas1r2_ (n = 11) and L_Tas1r2_ (n = 10) groups, as in the chow-fed BXD panel mice (Figure 4B, Supplemental File S1K) and confirmed the two groups to be statistically distinct (Figure 4C). BXD_HFD_ Tas1r2 correlates with Tas1r3, yet there is no statistical difference in Tas1r3 expression between the BXD_HFD_ H_Tas1r2_ and L_Tas1r2_ groups (Figure S2A,B). The GSEA analysis revealed that the BXD_HFD_ L_Tas1r2_ mice retained the enrichment of cytoplasmic ribosomal proteins, ETC, and OxPhos—mirroring chow-fed BXD L_Tas1r2_ and BL6 KO_Tas1r2_ muscle signatures (Figure 4D,E, Supplemental File S1L, Supplemental File M, Figure S2C). However, the BXD_HFD_ L_Tas1r2_ mice also exhibited the upregulation of pathways related to proteasome degradation, oxidative stress, and interleukin signaling, similar to BL6 KO_Tas1r2_ (Figure 4D). We then investigated the differences in the transcriptional regulation of key enriched pathways between the chow-fed and HFD conditions. The Pearson’s correlations of muscle-specific TFs and genes from the cytoplasmic ribosome and ETC-OxPhos revealed significant shifts between BXD_HFD_ and BXD within each Tas1r2 expression group (H_Tas1r2_ and L_Tas1r2_) (Figure 4F,G, Supplemental File S1N). Most of the significant correlation changes between the BXD_HFD_ and BXD mice occurred in the H_Tas1r2_ group, whereas the L_Tas1r2_ mice showed relatively fewer significant shifts. These results suggest that low Tas1r2 expression may buffer against the transcriptional reprogramming of the cytoplasmic ribosome and ETC-OxPhos pathways under metabolic stress, thereby preserving pathways associated with protein synthesis and mitochondrial function.

## 4. Discussion

Metabolic homeostasis relies on the ability of cells to sense and respond to nutrient availability, a process mediated in part by specialized receptors that regulate downstream signaling pathways. While the sweet taste receptor Tas1r2 is traditionally associated with gustatory perception, emerging evidence suggests a broader role in peripheral metabolism [15] including in the skeletal muscle [31,33], a key organ for energy sensing and metabolic adaptation. Understanding whether Tas1r2 influences muscle transcriptional networks could provide novel insights into how nutrient-sensing mechanisms integrate with metabolic regulation at the tissue level.

In this study, we leveraged the genetically diverse BXD mouse panel to characterize Tas1r2 expression in skeletal muscle and identify its transcriptional correlates under baseline and high-fat diet conditions. Our findings reveal that Tas1r2 expression varies widely across strains and is significantly associated with the gene networks involved in ribosomal activity, mitochondrial function, and lipid metabolism. Notably, low Tas1r2 expression in the muscles of BXD panel strains was associated with the upregulation of pathways linked to oxidative phosphorylation and protein synthesis, a pattern that was strikingly similar to that observed in muscles from C57BL/6J Tas1r2 knockout mice. This convergence suggests that reductions in Tas1r2 expression—even in the absence of complete gene deletion—may be sufficient to drive distinct metabolic adaptations in muscle. Since complete LOF variants in TAS1R2 are rare in humans, our findings support the idea that natural variation in gene expression levels or function could still have physiologically meaningful effects. Understanding these graded responses provides a framework for studying TAS1R2 function in human metabolism, where genetic polymorphisms that alter expression rather than abolish function are more common.

The BXD RI mouse strains, derived from crosses between C57BL/6J and DBA/2J progenitors, serve as a powerful resource for investigating the genetic underpinnings of complex traits, including transcriptional regulation in skeletal muscle. By combining the genetic diversity inherent in these strains with extensive phenotypic data, researchers can dissect the intricate relationships between genotype and phenotype [9]. The GeneNetwork.org database facilitates this process by providing a platform to integrate and analyze such multifaceted data. BXD panel mice have been instrumental in addressing diverse biological questions such as neurotoxicity mechanisms [45], or elucidating complex behavioral traits [46]. A notable example is the expansion of the BXD family to 140 fully isogenic strains, enhancing their utility in precision medicine. This expanded panel, characterized by over six million common DNA variants, allows for the high-power and precise mapping of heritable traits [8].

Several studies have leveraged the BXD panel to explore transcriptional regulation in skeletal muscle. For instance, a study examining cardiac α-actin (Actc1) expression in early adult skeletal muscles across BXD panel strains revealed significant variability. This variability was linked to strain-dependent methylation patterns around the Actc1 transcriptional start site, offering insights into the genetic regulation of Actc1 expression [47]. Similarly, the genetic determinants influencing the weight of fast- and slow-twitch skeletal muscles using 25 BXD panel strains was investigated [48]. The study revealed significant genetic effects on muscle weight, suggesting a polygenic origin for these traits, and highlighted the potential of BXD panel strains in dissecting the genetic basis of muscle phenotypes. In another study, BXD panel strains were utilized to identify candidate genes associated with skeletal muscle traits. By integrating RNA expression data with quantitative trait loci (QTL) mapping, they pinpointed genes that may influence muscle function and morphology. This integrative approach underscored the utility of BXD panel strains in linking genetic variation to gene expression and phenotypic outcomes [49].

Our study identified a significant overlap in transcriptional patterns between BXD L_Tas1r2_ mice and Tas1r2 KO (BL6 KO_Tas1r2_) mice, reinforcing the functional relevance of Tas1r2 expression levels in skeletal muscle. Despite differences in the genetic background and the nature of Tas1r2 deficiency—where BXD L_Tas1r2_ represents natural variation and the KO model represents complete gene deletion—the convergence in differentially expressed TFs suggests that reduced or absent Tas1r2 expression engages similar regulatory mechanisms. Among the shared TFs between BXD L_Tas1r2_ and BL6 KO_Tas1r2_ mice, we observed key regulators of metabolic and structural pathways. These factors exhibited consistent shifts in expression and correlation patterns, particularly within pathways related to cytoplasmic ribosomes and ETC-OxPhos, suggesting a coordinated regulatory response to Tas1r2 deficiency. Mlxipl (MLX interacting protein-like, also known as ChREBP) is a key regulator of glycolytic and lipogenic genes, influencing energy metabolism and mitochondrial biogenesis [50]. Nfic (Nuclear factor 1 C-type) is involved in cellular differentiation and has been implicated in the regulation of muscle-specific genes [51]. Rxrb (Retinoid X receptor beta) forms heterodimers with other nuclear receptors to modulate the gene expression linked to lipid metabolism and mitochondrial function [52]. Pbx2 (Pre B cell leukemia homebox 2) is a transcription factor that interacts with other proteins to regulate developmental processes and may influence muscle regeneration and repair [53]. Alterations in these transcription factors could disrupt the balance between protein synthesis and degradation, affecting muscle mass and function. Indeed, the muscle-specific deletion of Tas1r2 in mice (mKO) led to increased mitochondrial density and function [33]. Consequently, not only did mKO mice display increased running endurance [33], but they also demonstrated enhanced improvements in their mitochondrial content and running capacity compared to WT mice following an exercise training program [31]. mKO mice also displayed increased lean mass following the exercise training compared to their WT counterparts, which showed no changes [31]. Despite these shared signatures, the comparison between the BXD L_Tas1r2_ and BL6 KO_Tas1r2_ models is nuanced by methodological differences. The statistical power of the differential expression analysis in the BL6 KO_Tas1r2_ mice was constrained by a small sample size (n = 3/group), whereas the BXD dataset (n = 12/group) provided greater confidence in pathway enrichment. As a result, discrepancies in the differential expression of TFs between the models may reflect the underpowered BL6 KO_Tas1r2_ analysis, rather than fundamental biological differences. Furthermore, the gradient of Tas1r2 expression in BXD panel mice allows for low-confidence estimates of expression directionality in genes with insignificant differential expression, emphasizing the need for cautious interpretation.

Notably, our analysis revealed a negative correlation between Tas1r2 expression and several genes involved in fatty acid metabolism, including Acaa2, Dbi, Mdh1, Acadl, Acadm, Cyp7a1, Slc27a1, Ech1, Dld, Pgk2, Decr1, and Acat1. These genes encode enzymes and transporters essential for fatty acid oxidation and lipid handling in skeletal muscle [54]. For instance, Acaa2 encodes acetyl-CoA acyltransferase 2, a mitochondrial enzyme involved in the final step of the β-oxidation pathway, while Acadl and Acadm encode long-chain and medium-chain acyl-CoA dehydrogenases, respectively, which catalyze the initial dehydrogenation step in fatty acid β-oxidation. The observed negative correlation suggests that reduced Tas1r2 expression may be associated with enhanced fatty acid oxidation which is consistent with the mKO phenotype [31,33] and the pronounced transcriptional enrichment of ETC-OxPhos pathways in null and low Tas1r2 expressing muscles. These findings also underscore the potential compensatory or adaptive mechanisms that arise in response to diminished Tas1r2 signaling. The identification of overlapping TFs and pathway disruptions provides a foundation for further investigations into how nutrient-sensing GPCRs influence skeletal muscle metabolism and adaptation to metabolic stressors.

To shed light on how Tas1r2 influences adaptive responses to metabolic stress we analyzed muscle transcriptomics in HFD-fed BXD panel mouse strains. The BXD_HFD_ L_Tas1r2_ mice demonstrated the further enrichment of pathways related to proteasome degradation, oxidative stress, and interleukin signaling, aligning their transcriptomic profiles more closely with those observed in Tas1r2 whole-body knockout (bKO) models. This suggests that reduced or absent Tas1r2 expression may sensitize skeletal muscle to dietary-induced stress. In agreement, bKO mice exhibited enhanced metabolic efficiency, altered substrate utilization, and protection from hyperinsulinemia following HFD feeding [19], consistent with shifts in BXD_HFD_ L_Tas1r2_ muscle transcriptomics. Notably, HFD-fed bKO mice exhibited increased lean mass compared to their WT counterparts, despite similar gains in total body weight [19]. This further supports the role of Tas1r2 function in regulating muscle mass maintenance and underscores the complex interplay between genetic background, nutrient sensing, and metabolic adaptation. Our approach to analyzing differences in transcriptional regulation between chow and HFD conditions relied on shifts in TF correlations rather than direct ChIP-seq data. This strategy leveraged the statistical power of the BXD panel dataset to infer regulatory changes, but it also introduced limitations. Correlation-based analyses cannot establish causality or confirm regulatory interactions, emphasizing the need for future studies using complementary experimental techniques.

The observed transcriptomic similarities between BXD panel strains with low Tas1r2 expression and bKO (i.e., no Tas1r2 expression) models suggest that reduced Tas1r2 expression elicits molecular changes akin to complete gene knockout. This overlap implies that even partial LOF variants can significantly impact gene networks and pathways, leading to phenotypic outcomes comparable to those seen in knockout models. The BXD panel’s ability to exhibit a range of Tas1r2 expression levels, from high to low, provides a gradient of gene function that mirrors the spectrum of genetic variation found in humans. We have recently identified that a common SNP of TAS1R2 (Ile191Val) in humans [55] causes partial LOF, mirroring effects seen in LOF mouse models [28]. Similar to bKO mice, Val carriers demonstrated reduced glucose excursion following an oral glucose challenge [28]. Strikingly, Val carriers also displayed enhanced adaptations to exercise training, such as improved aerobic and muscle mitochondrial capacity, and enhanced muscle mass mirroring similar adaptations seen in muscle-specific LOF mouse models [31]. Therefore, the BXD panel strains are an effective dose effect model for exploring the effects of Tas1r2 expression, bridging the gap between total LOF knockout models (i.e., bKO and mKO) and partial LOF variants in humans (i.e., TAS1R2-Ile191Val). Such models are crucial for understanding how varying degrees of gene expression and function influence phenotype and disease susceptibility. Although we have confirmed TAS1R2 expression in cultured human myofibers isolated from muscle biopsies [31,33], publicly available resources such as the Human Protein Atlas and the GTEx database, report low or negligible TAS1R2 expression in human skeletal muscle. However, these same databases also report minimal or undetectable TAS1R2 expression in other tissues where its presence and function have been independently validated—such as the pancreas, small intestine, and whole blood [56,57,58]. This discrepancy suggests that current transcriptomic databases may underestimate TAS1R2 expression due to factors such as low transcript abundance, cell-type specificity, or technical limitations in detection sensitivity.

Nevertheless, our study has some notable limitations. While transcriptomic analysis provides insights into gene expression changes, we did not perform direct functional validation to confirm the causal role of the identified TFs and pathways in Tas1r2-mediated metabolic regulation. Although we infer functional compensation and regulatory convergence, particularly under high-fat diet conditions, these conclusions are based on correlation and would be strengthened by direct validation, such as proteomic profiling or metabolic assays. However, the BXD panel muscle transcriptomic datasets used in this study were obtained from GeneNetwork.org, and the original tissue samples are not available for follow-up experimentation. Likewise, proteomic data from BL6 Tas1r2 knockout mice are not currently available for comparisons. Moreover, we highlight several pathways related to lipid metabolism and oxidative stress, that require further in-depth mechanistic studies to clarify Tas1r2’s functional role in these processes. Our study focuses on skeletal muscle, but Tas1r2 is expressed in multiple peripheral tissues, suggesting effects that may be influenced by systemic interactions. While BXD panel strains provide a bridge between knockout models and human SNP carriers, species-specific differences in metabolic regulation and the artificially selected genetic diversity of the BXD panel may limit direct translational applications. Human studies are necessary to confirm whether the observed gene expression patterns and pathway activations hold in human skeletal muscle. Finally, while our study provides a comprehensive transcriptomic analysis, additional layers of regulation (e.g., epigenetic modifications, post-translational modifications, and protein activity) were not explored. Integrating multi-omics approaches, such as proteomics and metabolomics, could provide deeper mechanistic insights.

## 5. Conclusions

In summary, our study demonstrates that Tas1r2 influences skeletal muscle metabolism by modulating the transcriptional programs related to mitochondrial function, protein turnover, and lipid metabolism, which is aligned with phenotypic observations [33]. Under high-fat diet conditions, further enrichment in stress-related pathways suggests that Tas1r2 plays a role in adaptive metabolic reprogramming [59]. The transcriptomic overlap between the BXD L_Tas1r2_ and BL6 Tas1r2 knockouts demonstrates that the BXD panel models graded Tas1r2 loss, linking the metabolic effects of full gene deletion in mice to those of partial loss-of-function SNPs, like human Ile191Val.

## Data Availability

The transcriptomics data generated in this study have been deposited in the GEO repository under accession code GSE295025. The BXD skeletal muscle transcriptomics data (EPFL/LISP BXD CD+HFD Muscle Affy Mouse Gene 1.0 ST (Nov12) RMA Exon Level) was sourced from GeneNetwork.org.

## Supplementary Materials

The following supporting information can be downloaded at: https://www.mdpi.com/article/10.3390/nu17111918/s1, Figure S1: Transcriptional regulation of ETC-OxPhos in BL6 KO_Tas1r2_ and BXD L_Tas1r2_ muscles; Figure S2: Sweet taste receptor association and heatmap in BXD_HFD_ muscles; Supplemental File S1: Data Spreadsheet, tabs named A–N.

## Abbreviations

The following abbreviations are used in this manuscript:

LOF	Loss-of-function
SNP	Single nucleotide polymorphism
BXD	C57BL/6J × DBA/2J
RI	Recombinant Inbred
GRP	Genetic reference population
QTL	Quantitative trait locus
GWAS	Genome-wide association study
GPCR	G-protein coupled receptor
HFD	High-fat diet
KO	Knockout
WT	Wildtype
ORA	Over-representation analysis
OXPHOS	Oxidative Phosphorylation
GSEA	Gene Set Enrichment Analysis
ETC	Electron transport chain
TF	Transcription factor
